# Scraps

**Published:** 1899-12

**Authors:** 


### SCRAPS

PICKED UP BY THE ASSISTANT-EDITOR.

**Clinical Records** (29).—A woman whose husband had been a sufferer for many years wrote to the proprietors of a well-advertised preparation: "My husband had suffered terribly for a long time from a distressing complaint. After taking four doses of your medicine he was in quite another world."

**The Queen's Visit.**—In one of the Bristol wards notice was given that the entertainment provided by the Lord Mayor's Fund would be given to all those who were alive at the time of the Queen's accession. A woman who applied for a ticket said that she was born in November, 1837. When she was told that she was not eligible, as the Queen's accession was in June of that year, she asked: "Do you mean to say that I was not alive then?" The presiding official, whose embryology was sound, gave her a ticket.

**Obstetrics Home and Foreign** (7).—In the collection entitled Sylva, or the Wood, p. 130, we read that "a few years ago, in this same village, the women in labour used to drinke the urine of their husbands, who were all the while stationed, as I have seen the cows in St. James's Park, straining themselves to give as much as they can."—Brand's *Popular Antiquities*, ed. Bohn, 1849, vol. ii., pp. 69, 70.

In ancient Mexico, a decoction of the root of a plant called *civapaethi*, which possessed some oxytocic properties, was given, but if the pains were too severe a small piece of the tail of an opossum, carefully rubbed down in water, had to be taken. However ridiculous this may seem, it is not more so than a prescription given by the court physician in Siam to a lady of high rank at the time of her confinement: "Rub together shavings of sapan wood, rhinoceros blood, tiger's milk (a fresh deposit found on certain leaves in the forest), and cast-off skins of spiders."—Engelmann's *Labor Among Primitive Peoples*, 1882, p. 135.

**Medical Philology** (XXXII.).—The word "disease," now almost monopolised as a medical term, had formerly a much wider signification, "with distinct reference to the etymological elements of the word." In its non-medical meaning it has had a longer life as a verb than as a substantive.

Illustrating its wide range of signification, two instances may be quoted from the particularly interesting uses of the word given by the *New English Dictionary*. Where the Authorised Version reads in *John* xvi. 33, "In the world ye shall have tribulation," Wyclif (1388) has "In the world ȝe schulen haue disese," and in *Mark* v. 35 Tindale (1526) has "Thy doughter is deed; why deseasest thou the master eny further."

The *Catholicon* has "to Desese; *tedere*," with "to noye" in a cross-reference. Mr. Herrtage's note reads:

'*Desaise*, f. A sickenesse, a being ill at ease. *Desaisé*, out of temper, ill at ease.' Cotgrave. In the Version of the History of Lear and his daughters given in the *Gesta Romanorum*, p. 50, we are told how the eldest daughter, after keeping her father for less than a year, 'was so anoyed and *disessed* of hym and of his meanes' that she reduced the number of his attendants; and in chap. 45 we read of a law that the victor in battle should receive on the first day four honours, 'But the second day he shall suffre iiij. *diseases*, that is, he shall be taken as a theef, and shamfully ledde to the prison, and be dispoyled of Iubiter clothyng, and as a fole he shall be holden of all men; and so he shall have, thet went to the bataile, and had the victorie.' E. E. Text Soc. ed. Herrtage, p. 176.

**The Specialist and his Telegrams.**—Perhaps it is because the specialist is a modern product that his bent is so strongly towards modern ways, and towards utilising for his own purposes the facilities offered by modern social methods. That he should be a man of many instruments is not surprising, and that these instruments should run largely to electricity is only in the nature of things; that his letters should be typed and his notes dictated to a shorthand writer may be taken for granted; and that he should be on the telephone, and have a registered telegraphic address are mere matters of course. This telegraphic address is of peculiar interest, for it enables the specialist to do what without it he would find difficult; namely, to announce his line of practice. No one would care to put on his door-plate, "Specialist in Heart Disease," but nothing is simpler than to stamp one's notepaper, "Telegrams—'Cardiac, London,'" which serves the same purpose. Moreover, this is just the sort of thing the public likes. There is something so "simple" and "common sense" about it. It really is becoming a serious matter to the well-to-do public, who of course never write letters but telegraph for everything, to have to decide what doctor to call in! But this telegraphic address business is the very thing. Why bother about knowing the names of all the specialists? If one knows one's own complaint that is quite enough; and every person of intelligence, of course, knows what is the matter with him, notwithstanding the fuss that pedantic doctors make about what they call diagnosis! All that one has to do when one feels "chippy" is to decide what sort of a man one wants, and then to send for him. Is it headache that we suffer from? "Headache, London," will fetch us just the specialist we want. Or, if we feel doubtful about our liver, "Liver, London," will do the trick. Who the man is, who, as they say of dogs, "answers to the name," what he has done, or even what his real name is, we do not know; perhaps we do not care. Why, indeed, should we trouble about all these details? It is the specialist that we want, and by this ingenious device we get him. Perhaps the specialist also gets what he wants, so that the accommodation is quite mutual. Which ought to be satisfactory to all parties—except those who have been too late in choosing their registered telegraphic address.—*The Hospital.*

**Medicine as a Profession.**—In an address delivered to the candidates for graduation of the Johns Hopkins University, Dr. Walter B. Platt said: "As a physician you will change many of the ideas you have previously held to be true. One of these is the view you have held of your fellow-men. Men will be found to be much worse than you ever dreamed and much better than you ever supposed; stronger in right purpose than you believed, and failing those nearest them at the critical moment. You will more than accomplish the desire of Adam and become as a god, knowing good from evil more than other men. You will see the human heart stark naked, and beautiful or hideous, as it is made. No robe or cassock can hide the dreadful deformity which you alone of human beings will sometimes see beneath it. No station or high office of great estate will afford the least protection or shelter from your gaze. You will find hearts as dry of human kindness as any rock smitten by Moses in the wilderness was of water, but others brimming over with a constant and endearing sympathy that makes you instant captive. Your ideas of what men usually call the power of religion will be rudely shaken, for you will see men of years devoted to the observance of the churches tremble at the far-off bugle call of the gray angel, while gigantic rascals hear the near rustle of his wings without a tremor . . . You will believe as much in the perfect virtue of many good women as in the almost total depravity of others. At times you will think that all men are liars, until you find those whose perfect truthfulness a thousand times outweighs those dark and devious ways. To know how good and how bad men and women are, you must practise medicine. . . . To feel that you hold the life of a man in the hollow of your hand, that his earthly career is at the ends of your fingers, that you are surely going to save him from death by your action, by your intelligence, and that his years will go on as if nothing had dragged him to the edge of a precipice and almost over; is not this a triumph greater than that of a soldier, who has taken instead of saved human life? Can you imagine any greater satisfaction than to do this, not once only but many times, not to speak of the pleasure of relieving pain or bringing peace and comfort where there was unhappiness and discomfort. Every physician should have in his office, for his own edification, the finest obtainable copies of the 'Prodigal Son,' to see if art can equal what he daily sees and hears. Have a statue of Venus in an opposite corner from one of Cupid, for you are a daily witness of the trouble which comes to young men and old who follow strange gods. . . . A physician ought to be a gentle Stoic, a bit of an Epicurean, and a true Christian gentleman. We read that Arthur, when he made a knight, said: 'Be ye a good knight, and so I pray to God, so ye may be, and if ye be of prowess and of worthiness, ye shall be a knight of the Table Round'; and we who are physicians say this to every one of you."—*Boston Medical and Surgical Journal.*

**Medical Bibliography.**—By the recent death of the *Index Medicus* all medical libraries and workers in medicine have had a severe loss. In our June number a method was suggested for carrying on the work. Other schemes are in the air. At the last meeting of the American Medical Association it was resolved, on the motion of Dr. Gould, to whom medical literature owes so much: "That the executive committee of the American Medical Association appoint a committee of three members of the Association to take general charge of the publication of the periodical, perfect plans for the same, and engage the services of an editor and of such editorial assistants as may be required, etc., to choose a publisher and make contracts with him for the printing, distribution, etc., of the work, all in such a manner that it shall continue the high standard of accuracy and bibliographic usefulness so well established by the previous editors; and That the treasurer of the Association be instructed to pay all bills endorsed by said committee in payment of the necessary expenses of such editing and publication, provided that these do not exceed an annual outlay of over $3000." (*Journal of the American Medical Association*, June 10, 1899.) Dr. Marcel Baudouin, that prolific worker in so many departments of medicine, thinks that France is especially fitted to take a leading part in establishing a successor to the *Index Medicus*, because the recently-founded Institut de Bibliographie has a great deal of the material ready to hand. (*Gazette médicale de Paris*, 10 Juin, 26 Août, 1899. See also *Medical and Surgical* "*Review of Reviews*," July, 1899.) But whilst other people have been talking some persons in Vienna have been working, and several numbers of the *Index Medicus Novus* have been issued. That the interests of English readers will not be disregarded is evident from the circular which heralded the new venture, and of which I append a copy :—

" The necessity to have an organ of information of the whole medical literature of world is wellknowne percieved by all investigators and practioners all days more and more. Such work demands extreemely much toil, time and expenses, for there must be gathered the whole medical journal-literature of every country and even in all languishes, to give all times an resumption quite complete of the whole medical literature.

"Through the persuasion of prominent authorities and by cooperation of professioners, we resolved, to satisfy this pressing whishes, to produce such an underprize and so we founded the 'Index Medicus Novus.' — We must say that the sacrifices which are necessary to the work in each regard are more important as we thought in the beginn; but we will continue now the undertaken work because there is no doubt, that the whole medical world will have the greatest interest of it. Next time we will give also a specification of all medical-scientific-books coming just out, as all medical dissertations of the universities quite regular; so the 'Index Medicus Novus' will become a collection for consultation quite unsurpassed and as theer has been nothing likewise before it in the medical knowledge science.

" To have an easy and quiekly review of the great deal of the stuff we fixed to publish the 'Index Medicus Novus' twice mouthly to the 1. and 15., in mind to publish the literature of world who appeared in this short time as soon and complete as possible. Than we tryed to comprise the special compartments to have an easy review and we observe, that the division 'Naturalia' shows those essays which are composed by graduated phisicians or for the least acts of scientific, clinical questions and in the 'Academia' those recipients, which are discussed in the sessions of the academies of sciences on the jurisdiction for medical investigation. Those last named very important essays have been till now generally quite — unknown to the broader medical circles, because they are only published in the academical publications.

"Though all this performances we set the price for subscribition very low, to make it possible to each scientific searcher to buy the 'Index Medicus Novus.' So we hope confidently that the interested circles well assist (to this undertakement quite as important than scientific and exertions) by her participation and furtherance. We will not omit to say that our bureau translates in all languishes each essay wished by anyone.

" This number appears exeptionally as a double-number.

" We the honour to send you the 'Index Medicus Novus' and you can have, if you like it, still the numbers of the first quarter of the year.

" Saying that we will try to follow willingly to the wishes and incitations of learned-mans with great veneration

"the Redaction of

"INDEX MEDICUS NOVUS."

In order that an intending purchaser shall not go wrong, a detachable slip, worded in the form given below, is provided for the purpose of ordering the publication :

ORDRE.

The not disired is to strike through.

**To the redaction of "INDEX MEDICUS NOVUS"**
Vienna, I. Tuchlauben 23.

I subscribe the "**Index Medicus Novus**" from the 1. Oct. ....................... till ............................. and send you the amount of

Frs. 5.— for 1 quarter of a year

„ 10.— „ ½ year

„ 20.— „ 1 year

Please to give a very clear adress

Date

.......................................................................................................

The ordre is to rip and send by the post in enveloppe to the redaction of "Index Medicus Novus."

